# MiR-199a-3p deficiency induced by STAT3 activation drives smooth muscle cell phenotypic switching in pulmonary arterial hypertension

**DOI:** 10.1016/j.ncrna.2026.06.001

**Published:** 2026-07-09

**Authors:** Wen-Xia He, Yun-Jie Huang, Tian-Hong Ai, Song-Qun Huang, Xiao-Hua You, Hong Wu, Xiao-Wei Song

**Affiliations:** Department of Cardiology, Changhai Hospital, Second Military Medical University, Shanghai, China

**Keywords:** MiR-199a-3p, STAT3, Pulmonary arterial hypertension

## Abstract

Pulmonary arterial hypertension (PAH) is a progressive vasculopathy characterized by pathological vascular remodeling, in which pulmonary artery smooth muscle cells (PASMCs) undergo phenotypic switching from a contractile to a synthetic state, driving excessive proliferation and migration. Here, we report that miR-199a-3p is significantly downregulated in both hypoxia/SU5416-induced PAH rat models and PDGF-BB-stimulated human PASMCs. Mechanistically, PDGF-BB stimulation induces the phosphorylation of STAT3, which directly binds to the TTCCCGGAA motif within the promoter of miR-199a-3p. This binding transcriptionally represses miR-199a-3p. Functional analyses demonstrated that miR-199a-3p overexpression maintains the PASMC contractile phenotype (upregulating MYH11 and SM22α) and suppresses proliferation and migration. Conversely, miR-199a-3p inhibition promotes synthetic phenotypic switching and activates the ERK/AKT signaling pathway. Furthermore, we identified YAP1 as a novel direct target of miR-199a-3p, linking miRNA downregulation to downstream proliferative signaling. Collectively, these findings establish a PDGF-BB/STAT3/miR-199a-3p/YAP1 regulatory axis that drives pathological PASMC phenotypic switching. This axis represents a promising therapeutic target for mitigating pulmonary vascular remodeling in PAH.

## 1 Introduction

Pulmonary arterial hypertension (PAH), classified as Group 1 pulmonary hypertension, has a prevalence of 48-55 cases per million population in developed countries. Characterized by multifactorial pathogenesis and rapid disease progression, PAH imposes significant physical, social, occupational, and emotional burdens on affected patients and their caregivers [1]. The primary pathological features include progressive remodeling of pulmonary arterioles, sustained increases in pulmonary vascular resistance, and eventual right ventricular failure [2]. Pulmonary artery smooth muscle cells (PASMCs) exhibit marked plasticity, enabling dynamic phenotypic switching between contractile and synthetic states under specific conditions. The cellular phenotype can be determined by evaluating expression changes in key markers: MYH11 and SM22α (indicating a contractile phenotype) versus CyclinD1 and PCNA (indicating a synthetic phenotype) [3]. Critically, synthetic phenotype PASMCs serve as pivotal effector cells in pulmonary vascular remodeling [4].

The mitogen-activated protein kinase (MAPK) pathway, particularly the RAF-MEK-ERK cascade, regulates cell survival, proliferation, and differentiation [5]. Phosphorylated ERK (p-ERK) exerts dual roles: cytoplasmic activation of cytoskeletal and metabolic regulators, and nuclear modulation of transcription factors driving cell cycle progression [6]. Similarly, the phosphatidylinositol 3-kinase (PI3K)/AKT pathway, conserved across eukaryotes [7], governs growth, migration, and metabolism via AKT phosphorylation at Ser 473/Thr308. Both pathways are implicated in PASMC phenotypic switching and pulmonary vascular remodeling. Liu et al. [8] reported that bortezomib attenuates angiotensin II/PDGF-BB-induced PASMC proliferation by suppressing ROS-dependent ERK and AKT activation. Griffiths et al. [9] demonstrated enhanced AKT/ERK phosphorylation and a Warburg-like metabolic shift in PAH-PASMCs, reversible by perhexiline-mediated AKT inhibition. Li et al. [10] further identified Maresin1 as a suppressor of STAT3, AKT, ERK, and FoxO 1 phosphorylation, inhibiting PASMC proliferation and promoting apoptosis.

MicroRNAs (miRNAs) are small non-coding RNAs, approximately 22 nucleotides in length, that post-transcriptionally regulate gene expression. They bind to the 3′ untranslated region of target mRNAs, leading to translational repression or mRNA degradation [11]. Among miRNAs, miR-199a has been implicated in modulating cellular processes such as proliferation, migration, apoptosis, autophagy, and glucose metabolism [12]. Studies in aortic and carotid artery smooth muscle cells (VSMCs) suggest that miR-199a-3p suppresses inflammatory responses and inhibits proliferation and migration. González-López et al. [13] found significant downregulation of miR-199a-3p in apolipoprotein E-deficient (ApoE^−/−^) mice and human atherosclerotic plaques of the aorta and carotid arteries. Mechanistically, overexpression of miR-199a-3p in VSMCs reduces oxidized low-density lipoprotein uptake, inhibits NF-κB inflammatory pathway activation, and attenuates atherosclerosis progression. Sun et al. [14] later showed that miR-199a-3p suppresses early atherosclerosis by inhibiting VSMC proliferation and migration. Nevertheless, whether miR-199a-3p exerts similar regulatory effects in pulmonary artery smooth muscle cells (PASMCs) remains to be experimentally validated.

This study investigates the mechanism by which miR-199a-3p regulates pulmonary artery smooth muscle cell (PASMC) phenotypic switching. To achieve this, a rat pulmonary arterial hypertension (PAH) model was established using hypoxia combined with SU5416 treatment. Additionally, an *in vitro* human PASMC phenotypic switching model was employed. These models were used to investigate miR-199a-3p expression changes in both *in vivo* and *in vitro* settings. Bioinformatics databases and literature mining were utilized to predict transcription factors regulating DNM3os/miR-199a-3p biogenesis. The specificity of transcription factor binding to the DNM3os promoter was validated using dual-luciferase reporter assays. Functional effects of miR-199a-3p overexpression and knockdown on PASMC phenotypic modulation were assessed, followed by an initial investigation of downstream signaling pathways.

## 2 Matetials and methods

### 2.1 Cells and reagents

Human pulmonary artery smooth muscle cells (hPASMCs) were obtained from Lonza (USA). SMCM medium, supplemented with smooth muscle cell growth supplement (SMCGS) and penicillin/streptomycin (P/S), was sourced from ScienCell Research Laboratories (USA). Reverse transcription kits were purchased from TOYOBO (cat# FSQ-101, 326,600, Japan). Quantitative real-time PCR reagents were sourced from Tiangen Biotech (cat# L0831, China). Protein extraction and quantification kits were obtained from Beijing TransGen Biotech (China). Lipofectamine RNAiMAX transfection reagent was purchased from Invitrogen (USA). CCK-8 reagent was acquired from Beyotime Biotechnology (Shanghai, China). Transwell chambers were purchased from Merck Millipore (Germany). Primers for DNM3os, PCNA, SM22α, STAT3, β-actin, U6, and miR-199a-3p were synthesized by GENEWIZ (Suzhou, China). NC (negative control), miR-199a-3p mimic, and miR-199a-3p inhibitor were obtained from GenePharma (Suzhou, China). Antibodies against PCNA (BM0104) and SM22α (BM5784) were procured from BOSTER (China). Antibodies targeting POLR2A (A2107), p-POLR2A (AP0997), STAT3 (A19566), p-STAT3 (AP0715), RAD21 (A19749), ATF2 (A0757), p-ATF2 (AP0523), and MYH11 (A10827) were obtained from Abclonal (China). Antibodies for GAPDH (AB0037), P38 (CY5488), p-P38 (CY8114), p-AKT (CY6017), and secondary antibodies (goat anti-rabbit IgG-HRP, AB0101; goat anti-mouse IgG-HRP, AB0102) were purchased from Abways (China). ERK1/2 (11257-1-AP) and p-ERK1/2 (80031-1-RR) antibodies were sourced from Proteintech (USA).

### 2.2 Cell culture and treatment

PASMCs were cultured in SMCM complete medium supplemented with fetal bovine serum (FBS), growth factors, and antibiotics at 37°C in a 5% CO_2_ incubator. For serum starvation, cells were maintained in serum-free medium for 12 h, followed by treatment with PDGF-BB (20 ng/mL) for 48 h. Cells were divided into three groups: negative control (NC), miR-199a-3p mimic-transfected, and miR-199a-3p inhibitor-transfected. Transfected cells were incubated for 48 h before other experiments.

### 2.3 Quantitative real-time PCR

Total RNA was extracted using Trizol reagent, and cDNA was synthesized via reverse transcription kit. RT-QPCR was performed using SYBR Green I-containing reagents and gene-specific primers on a LightCycler 480 system (Roche, Switzerland). U6 and β-actin served as internal controls for miR-199a-3p and DNM3os/PCNA/SM22α/STAT3, respectively. Relative expression was calculated using the 2^−ΔΔCT^ method.

### 2.4 Western blot

Cellular proteins were extracted, and concentrations were quantified using a BCA assay. Equal protein amounts were separated by SDS-PAGE and transferred to PVDF membranes. Membranes were blocked with 5% skim milk for 2 h, incubated with primary antibodies (1:1000 dilution; except GAPDH at 1:5000) overnight at 4°C, washed with TBST, and incubated with HRP-conjugated secondary antibodies (1:3000) for 2 h at room temperature. Bands were visualized using chemiluminescence, and grayscale analysis was normalized to GAPDH.

### 2.5 CCK-8 proliferation assay

Cells were resuspended at 2.5 × 10^4^/mL, seeded into 96-well plates (100 μL/well), and cultured for 24 h, 48 h, or 72 h. CCK-8 reagent (10 μL/well) was added, and after 2 h incubation, absorbance at 450 nm was measured using a Thermo Fisher microplate reader.

### 2.6 Scratch wound healing assay

PASMCs were seeded in 6-well plates until 95% confluent. Two perpendicular scratches were made using a 1 mL pipette tip. After PBS washes, cells were cultured in serum-free medium. Wound healing was monitored at 0 h, 24 h, and 36 h using an inverted microscope imaging system.

### 2.7 Transwell migration assay

Cells (2.5 × 10^5^/mL in serum-free medium) were added to Transwell chambers (200 μL/insert) placed in 24-well plates containing 600 μL complete medium with 15% FBS. After 36 h, non-migrated cells inside the chamber were removed with a cotton swab. Migrated cells underneath the chamber were fixed with 4% paraformaldehyde, stained with 0.1% crystal violet, and counted in three random fields under a microscope.

### 2.8 Dual-luciferase reporter assay

To validate the direct binding of STAT3 to miR-199 promoter and confirm YAP1 as a direct target of miR-199a-3p, dual-luciferase reporter assays were performed as follows. The wild-type (WT) miR-199 promoter fragment containing the predicted TTCCCGGAA STAT3 binding motif was amplified and cloned into the psi-promoter vector (JunFu Shanghai) upstream of the firefly luciferase gene. A mutant-type (MUT) plasmid was generated using a site-directed mutagenesis kit, altering the core binding sequence to disrupt STAT3 interaction. The 3′-untranslated region (3′UTR) of YAP1 containing the putative miR-199a-3p binding site was amplified and inserted downstream of the firefly luciferase gene in psiCheck2 vector (Promega). HEK-293 T cells were used for transfection. For promoter assays, cells were co-transfected with WT or MUT miR-199 promoter plasmid and STAT3 overexpressing or control plasmids. For miRNA targeting assays, cells were co-transfected with *YAP1* 3′UTR plasmid and miR-199a overexpressing or control plasmids. 24 h post-transfection, cells were washed with PBS and lysed using Passive Lysis Buffer. Luciferase activities were measured using the Dual-Luciferase® Reporter Assay System (Promega) according to the manufacturer's protocol. Firefly luciferase activity (experimental reporter) was measured first, followed by *Renilla* luciferase activity (internal control) after quenching. Luminescence was detected using a GloMax® 20/20 Luminometer. The relative luciferase activity was calculated as the ratio of Firefly luciferase activity to *Renilla* luciferase activity.

### 2.9 Statistical analysis

All experiments were performed in triplicate. Data are displayed as mean ± standard deviation (SD). Statistical analyses were proceeded using GraphPad Prism software version 8.0 (GraphPad Software Inc., USA). Paired comparisons were computed using paired *t*-test or repeated measures ANOVA, with *p* value less than 0.05 indicating significance.

### 2.10 Animal experiments

Eight-week-old male Sprague-Dawley (SD) rats (approximately 200 g) were randomly assigned to a control group (*n* = 3) and an experimental group (*n* = 3). To induce pulmonary arterial hypertension, rats in the experimental group received a weekly subcutaneous injection of SU5416 (10 mg/kg in DMSO) and were subjected to hypoxic conditions (10% O₂) for 12 hours daily over a period of 3 weeks. The control group received vehicle injections (DMSO) and were maintained under normoxic conditions. At the end of the 3-week protocol, all animals were anesthetized with isoflurane (1.5–2.5%), and heart and lung tissues were harvested for subsequent analysis. All animal procedures were conducted in strict accordance with the guidelines approved by the Institutional Animal Care and Use Committee of the Naval Medical University (Approval No. 8237021375, Date: 2023-03-08).

## 3 Results

### 3.1 MiR-199a-3p is consistently downregulated in PAH patient PASMCs, Hypoxia/SU5416 rat model, and PDGF-BB-induced phenotypic switching model

**Fig. 1:**
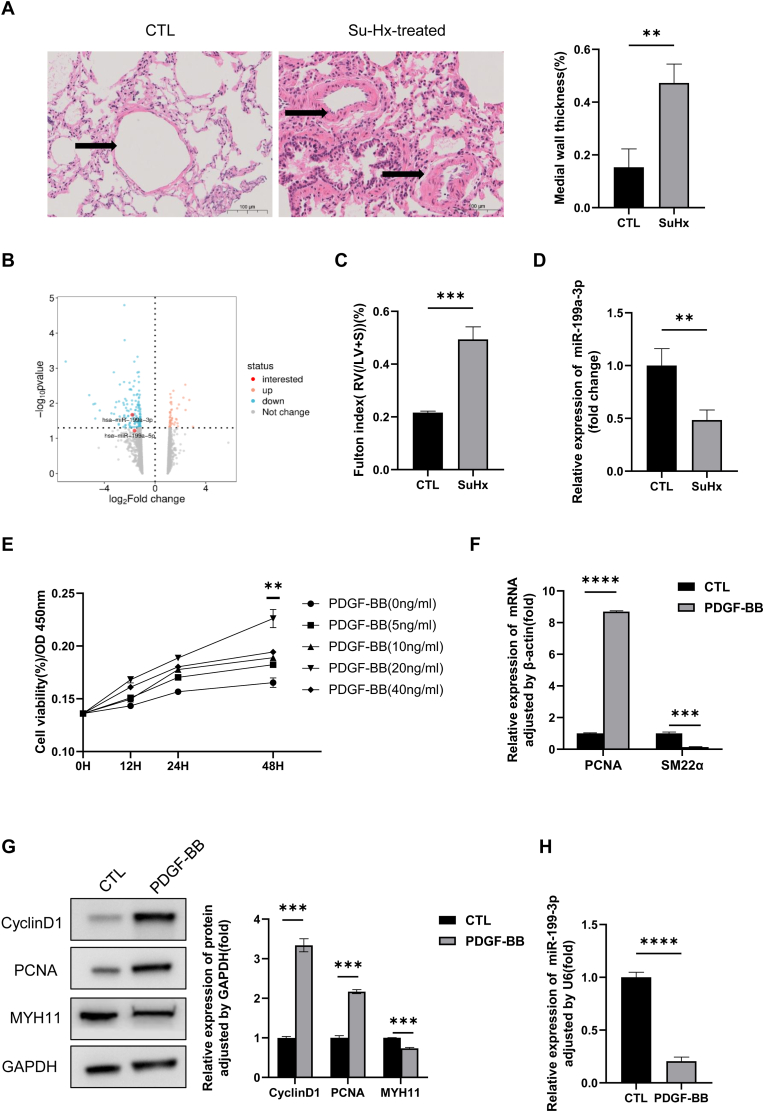
**MiR-199a-3p is downregulated in PAH models and PDGF-BB-treated hPASMCs.** (A) HE staining of pulmonary arteries showing increased wall thickness in SuHx-treated PAH rats versus Control (CTL) rats. (B) Volcano plot of miRNA microarray data (GSE108707) demonstrating significant downregulation of miR-199a-3p in PASMCs from PAH patients (n = 6) compared to healthy donors (n = 3). (C) Right ventricular hypertrophy index (RV/[LV + S]) in Control and SuHx-treated PAH rats. (D) qRT-PCR analysis showing reduced miR-199a-3p expression in lung tissues of SuHx-treated PAH rats versus Controls. (E) CCK-8 assay revealing dose-dependent PDGF-BB-induced proliferation of hPASMCs (maximal at 20 ng/mL). (F) qRT-PCR analysis of phenotypic marker mRNA levels (PCNA, SM22α) in hPASMCs treated with 20 ng/mL PDGF-BB for 48 h. (G) Western blot analysis of phenotypic marker protein levels (CyclinD1, PCNA, MYH11) in PDGF-BB-treated hPASMCs. (H) qRT-PCR analysis confirming miR-199a-3p downregulation in PDGF-BB-treated hPASMCs. ∗∗*p* < 0.01, ∗∗∗*p* < 0.001, ∗∗∗∗*p* < 0.0001.

Analysis of miRNA microarray data (GSE108707) from the GEO database revealed significant downregulation of miR-199a-3p (but not miR-199a-5p) in PASMCs from PAH patients (n = 6) compared to healthy controls (n = 3), as illustrated by the volcano plot (Fig. 1B). In the hypoxia/SU5416-induced rat PAH model, HE staining of right lung tissue demonstrated marked thickening of pulmonary arterial walls in the experimental group compared to controls (Fig. 1A). The right ventricular weight ratio (RV/[LV + S]) was significantly elevated (Fig. 1C). RT-qPCR analysis confirmed pronounced downregulation of miR-199a-3p in lung tissues of PAH rats versus controls (Fig. 1D). In the *in vitro* model, treatment of hPASMCs with PDGF-BB (0-40 ng/mL) induced dose-dependent increases in proliferation, with the maximal effect observed at 20 ng/mL (Fig. 1E). Stimulation with 20 ng/mL PDGF-BB for 48 h significantly upregulated mRNA levels of the synthetic marker PCNA while downregulating the contractile marker SM22α (Fig. 1F). Western blot analysis further confirmed elevated protein levels of CyclinD1 and PCNA, alongside reduced levels of MYH11 (Fig. 1G). Correspondingly, miR-199a-3p expression was significantly downregulated in PDGF-BB-treated hPASMCs (Fig. 1H).

### 3.2 PDGF-BB activates STAT3 to bind the DNM3os promoter at TTCCCGGAA region, suppressing transcription and miR-199a-3p expression

**Fig. 2:**
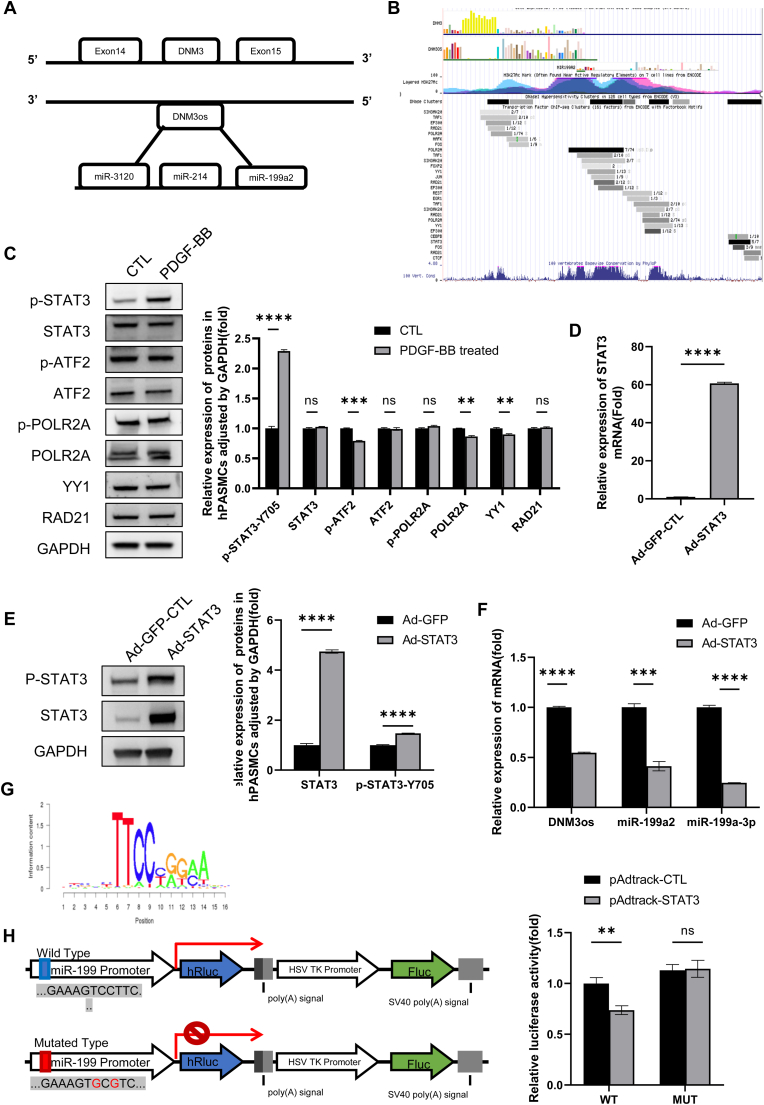
**STAT3 transcriptionally represses DNM3os and miR-199a-3p.** (A) Genomic localization of miR-199 within the *DNM3os* primary transcript. (B) ENCODE database prediction of transcription factors (TFs) binding the *DNM3os* promoter. (C) Western blot analysis of candidate TF protein levels in PDGF-BB-treated hPASMCs (20 ng/mL, 48 h), showing selective upregulation of phosphorylated STAT3 (p-STAT3). (D) qRT-PCR analysis confirming increased *STAT3* mRNA expression in hPASMCs transduced with Ad-STAT3 versus Ad-GFP control. (E) Western blot analysis demonstrating elevated STAT3 and p-STAT3 protein levels in Ad-STAT3-transduced hPASMCs. (F) qRT-PCR analysis showing reduced expression of *DNM3os*, miR-199a2, and miR-199a-3p in Ad-STAT3-transduced hPASMCs. (G) Conserved STAT3 binding motif (TTCCCGGAA) identified within the *DNM3os* promoter region by Cistrome and JASPAR analyses. (H) Dual-luciferase reporter assay demonstrating STAT3-mediated suppression of wild-type (WT) *DNM3os* promoter activity, which is abolished by mutation (Mut) of the STAT3 binding motif (TTCC→TGCG). ∗∗*p* < 0.01, ∗∗∗*p* < 0.001, ∗∗∗∗*p* < 0.0001.

Analysis of the NCBI database localized the miR-199a-3p precursors (miR-199a2) to the *DNM3os* locus, which also harbors miR-214 and miR-3120 (Fig. 2A). Screening of the ENCODE database predicted STAT3, RAD21, ATF2, POLR2A, and YY1 as potential transcription factors regulating *DNM3os* transcription (Fig. 2B). Among these candidates, PDGF-BB stimulation selectively upregulated the levels of phosphorylated STAT3 (p-STAT3) in hPASMCs (Fig. 2C). Adenovirus-mediated overexpression of STAT3 in hPASMCs increased both total STAT3 and p-STAT3 protein levels (Fig. 2D and E), while significantly reducing the expression of *DNM3os*, miR-199a-3p, and miR-199a2 (Fig. 2F). To identify the specific binding site, Cistrome and JASPAR analyses were performed, revealing a conserved STAT3-binding motif (TTCCCGGAA) within the *DNM3os* promoter region (Fig. 2G). We generated a site-directed mutant of this motif (TTCC mutated to TGCG). Dual-luciferase reporter assays demonstrated that STAT3 overexpression suppressed luciferase activity driven by the wild-type *DNM3os* promoter, but had no effect on the activity driven by the mutated promoter construct (Fig. 2H). These results indicate that STAT3 binds specifically to the TTCCCGGAA motif to transcriptionally repress *DNM3os*.

### 3.3 Overexpression of miR-199a-3p suppresses synthetic phenotype switching in hPASMCs

**Fig. 3:**
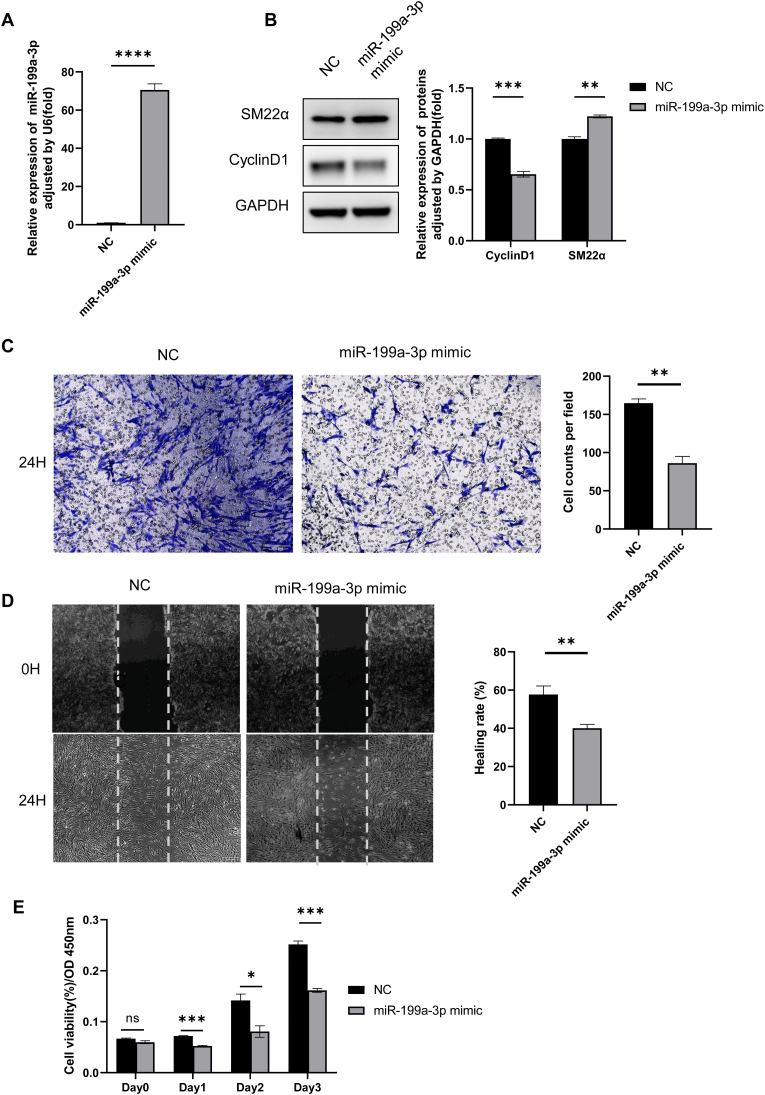
**MiR-199a-3p overexpression inhibits synthetic phenotypic switching in hPASMCs.** (A) qRT-PCR analysis confirming successful transfection and increased miR-199a-3p expression in hPASMCs transfected with miR-199a-3p mimic versus mimic NC (negative control). (B) Western blot analysis showing downregulation of the synthetic marker Cyclin D1 and upregulation of the contractile marker SM22α protein levels following miR-199a-3p mimic transfection. (C-D) Scratch wound healing and Transwell migration assays revealing significantly inhibited migration capacity in hPASMCs overexpressing miR-199a-3p. (E) CCK-8 assay demonstrating significantly suppressed proliferation of miR-199a-3p-overexpressing hPASMCs from Day 1 to Day 3. ∗*p* < 0.05, ∗∗*p* < 0.01, ∗∗∗*p* < 0.001, ∗∗∗∗*p* < 0.0001.

Transfection of hPASMCs with the miR-199a-3p mimic significantly increased miR-199a-3p expression, as confirmed by RT-qPCR (Fig. 3A). Consistent with a shift towards the contractile phenotype, Western blot analysis showed reduced protein levels of the synthetic marker CyclinD1 and elevated levels of the contractile marker SM22α (Fig. 3B). Furthermore, scratch wound healing and Transwell migration assays demonstrated that miR-199a-3p overexpression significantly inhibited hPASMC migration (Fig. 3C and D). Finally, CCK-8 proliferation assays revealed a marked decrease in optical density (OD) values from Day 1 to Day 3 in the overexpression group compared to controls, indicating suppressed proliferative capacity (Fig. 3E).

### 3.4 Knockdown of miR-199a-3p promotes synthetic phenotype switching in hPASMCs

**Fig. 4:**
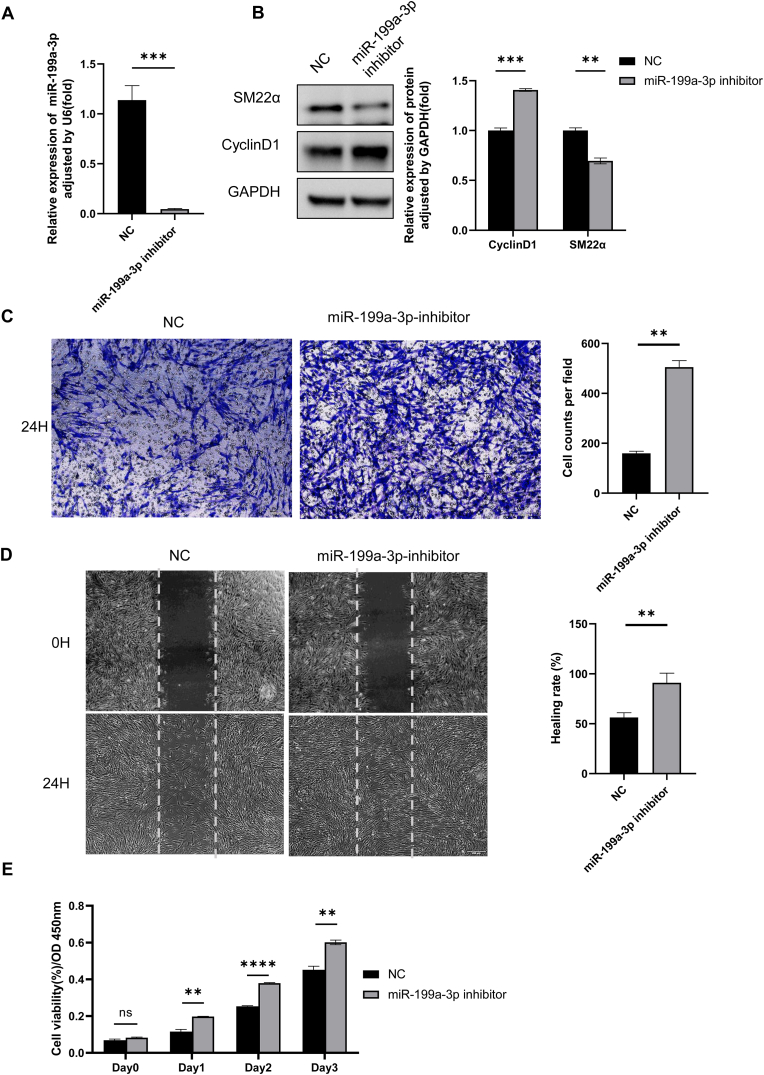
**MiR-199a-3p knockdown promotes synthetic phenotypic switching in hPASMCs.** (A) qRT-PCR analysis confirming successful transfection and reduced miR-199a-3p expression in hPASMCs transfected with miR-199a-3p inhibitor versus inhibitor NC (negative control). (B) Western blot analysis showing upregulation of the synthetic marker Cyclin D1 and downregulation of the contractile marker SM22α protein levels following miR-199a-3p inhibitor transfection. (C-D) Scratch wound healing and Transwell migration assays revealing significantly increased migration capacity in hPASMCs with miR-199a-3p knockdown. (E) CCK-8 assay demonstrating significantly enhanced proliferation of miR-199a-3p-knockdown hPASMCs from Day 1 to Day 3. ∗∗*p* < 0.01, ∗∗∗*p* < 0.001, ∗∗∗∗*p* < 0.0001.

Transfection of hPASMCs with the miR-199a-3p inhibitor significantly reduced miR-199a-3p expression, as confirmed by RT-qPCR (Fig. 4A). Consistent with a shift towards the synthetic phenotype, Western blot analysis showed increased protein levels of the synthetic marker CyclinD1 and decreased levels of the contractile marker SM22α (Fig. 4B). Furthermore, scratch wound healing and Transwell migration assays demonstrated that miR-199a-3p knockdown significantly accelerated hPASMC migration (Fig. 4C and D). Finally, CCK-8 proliferation assays revealed elevated optical density (OD) values from Day 1 to Day 3 in the knockdown group compared to controls, reflecting enhanced proliferative capacity (Fig. 4E).

### 3.5 MiR-199a-3p suppressed MAPK/ERK and PI3K/AKT signaling pathways

**Fig. 5:**
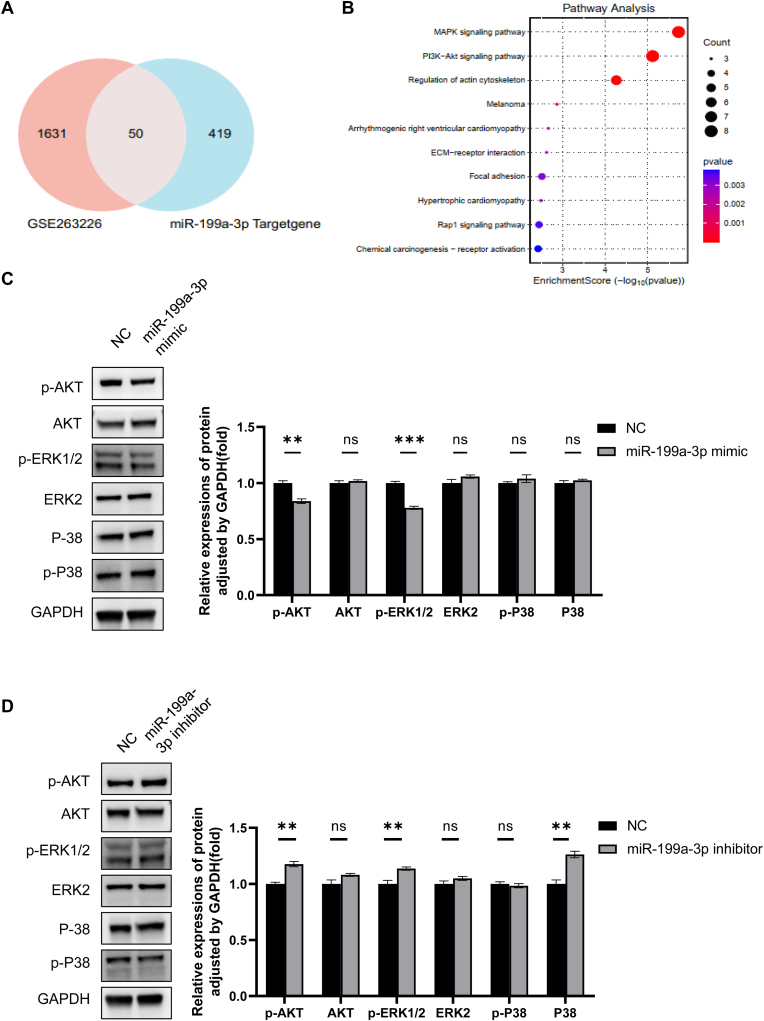
**MiR-199a-3p inhibits PASMC phenotypic switching by suppressing MAPK/ERK and PI3K/AKT signaling.** (A) Venn diagram showing overlap between differentially expressed genes (DEGs) in PAH-PASMCs (vs. healthy controls; n = 5 vs. n = 4; GSE263226) and predicted miR-199a-3p target genes (TargetScan), identifying 50 common genes. (B) KEGG pathway enrichment analysis of the 50 overlapping genes revealing significant enrichment in MAPK and PI3K-AKT signaling pathways. (C) Western blot analysis demonstrating that miR-199a-3p overexpression significantly suppresses phosphorylation of ERK and AKT in hPASMCs, indicating inhibition of MAPK/ERK and PI3K/AKT pathways. (D) Western blot analysis showing that miR-199a-3p knockdown enhances phosphorylation of ERK and AKT, activating MAPK/ERK and PI3K/AKT pathways. ∗∗*p* < 0.01, ∗∗∗*p* < 0.001.

Analysis of RNA sequencing data from PASMCs of PAH patients (n = 5) versus healthy controls (n = 4), retrieved from the GEO database (GSE263226), identified 1681 differentially expressed genes (DEGs) in PAH-PASMCs. Intersecting these DEGs with predicted miR-199a-3p target genes (469 genes from TargetScan) yielded 50 overlapping genes (Fig. 5A). KEGG enrichment analysis of these overlapping genes revealed the MAPK and PI3K/AKT pathways as significantly enriched signaling cascades (Fig. 5B). To functionally validate these findings, Western blot analysis was performed. Overexpression of miR-199a-3p significantly suppressed the phosphorylation levels of ERK and AKT, indicating inhibition of both the MAPK/ERK and PI3K/AKT pathways (Fig. 5C). Conversely, knockdown of miR-199a-3p enhanced ERK and AKT phosphorylation, demonstrating activation of these pathways (Fig. 5D).

### 3.6 MiR-199a-3p directly targets YAP1 and modulates its phosphorylation status

**Fig. 6:**
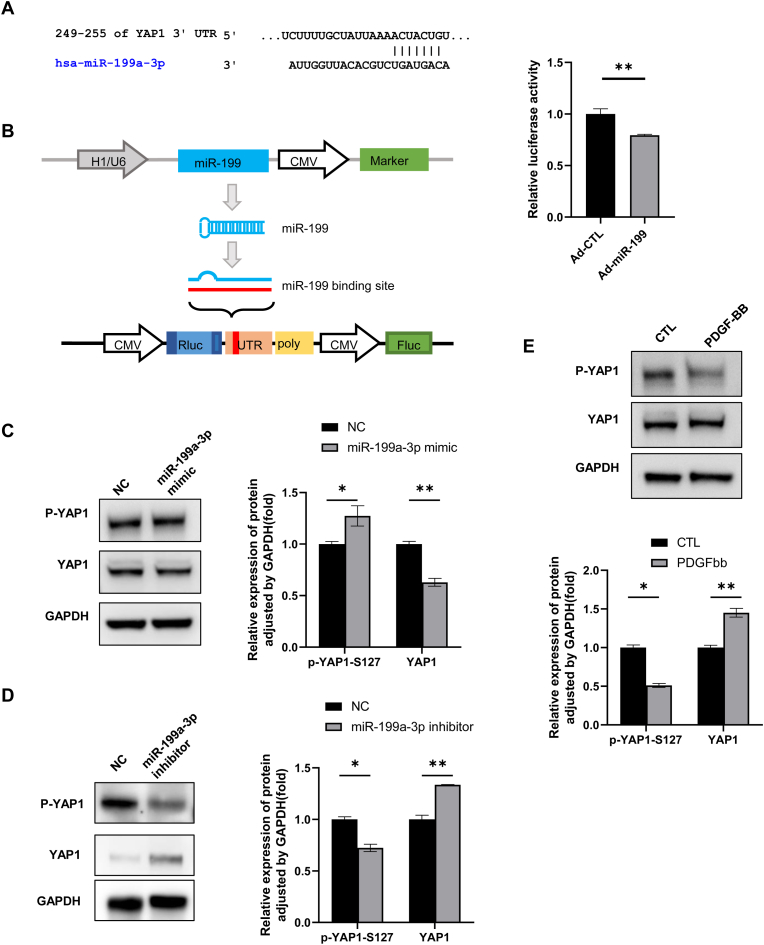
**MiR-199a-3p directly targets and suppresses YAP1 expression.** (A) Schematic illustration of the predicted binding site for miR-199a-3p within the 3′-untranslated region (3′UTR) of YAP1 mRNA (nucleotides 249-255). (B) Dual-luciferase reporter assay showing that co-transfection with miR-199a-3p significantly reduced the luciferase activity of the reporter vector containing the wild-type YAP1 3′UTR compared to control (CTL), confirming direct binding. (C) Western blot analysis showing that overexpression of miR-199a-3p (mimic) in PASMCs significantly decreased total YAP1 protein levels and increased the phosphorylation of YAP1 at Serine 127 (p-YAP1-S127) compared to the NC group. (D) Knockdown of miR-199a-3p resulted in a significant upregulation of total YAP1 protein and a reduction in p-YAP1-S127 levels. (E) Treatment with PDGF-BB significantly increased total YAP1 expression and decreased p-YAP1-S127 levels compared to the control group, consistent with the downregulation of miR-199a-3p. ∗*p* < 0.05, ∗∗*p* < 0.01.

To investigate the molecular mechanism by which miR-199a-3p regulates PASMC function, we utilized bioinformatic algorithms to predict potential downstream targets. The analysis identified a conserved binding site for miR-199a-3p located at nucleotides 249–255 within the 3′-untranslated region (3′-UTR) of Yes-associated protein 1 (YAP1) mRNA (Fig. 6A). To validate this interaction, we performed dual-luciferase reporter assays. Co-transfection of miR-199a-3p with a luciferase reporter vector containing the wild-type YAP1 3′-UTR significantly suppressed luciferase activity compared to the negative control group (Fig. 6B), indicating that miR-199a-3p directly binds to the YAP1 3′-UTR. Subsequently, we examined the regulatory effect of miR-199a-3p on YAP1 protein expression and its phosphorylation status. Western blot analysis revealed that overexpression of miR-199a-3p via mimics significantly downregulated total YAP1 protein levels. Concomitantly, the phosphorylation of YAP1 at Serine 127 (p-YAP1-S127) was markedly increased in the mimic group compared to the NC group (Fig. 6C). Conversely, knockdown of endogenous miR-199a-3p using specific inhibitors resulted in a significant upregulation of total YAP1 protein and a concomitant decrease in p-YAP1-S127 levels (Fig. 6D). Consistent with the *in vitro* findings, stimulation of PASMCs with PDGF-BB, which induces miR-199a-3p downregulation, led to a significant increase in total YAP1 expression and a reduction in p-YAP1-S127 levels (Fig. 6E). Collectively, these data demonstrate that miR-199a-3p directly targets YAP1 to suppress its expression and promote its inhibitory phosphorylation.

**Fig. 7:**
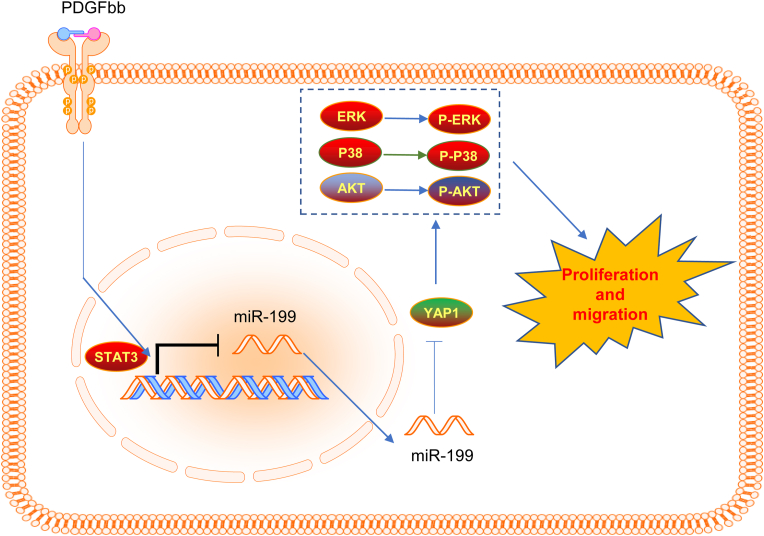
**The PDGF-BB/STAT3/miR-199a-3p/YAP1 Axis Regulates PASMC Proliferation and Migration via ERK, p38, and AKT Signaling Pathways.** PDGF-BB signaling induces STAT3 phosphorylation, which transcriptionally suppresses miR-199a-3p. This downregulation leads to the derepression and accumulation of YAP1, a critical node that activates ERK, p38, and AKT cascades. Collectively, this axis drives PASMC proliferation and migration, promoting vascular remodeling.

Collectively, we propose a comprehensive signaling model illustrating the molecular mechanism underlying PASMC phenotypic switching (Fig. 7). Our data indicate that the pathological process is initiated by the stimulation of PDGF-BB, which activates its cognate receptor on the cell surface. This activation triggers the phosphorylation of the transcription factor STAT3, which subsequently translocates into the nucleus. Inside the nucleus, phosphorylated STAT3 acts as a transcriptional repressor of the miR-199 gene, leading to the downregulation of the mature microRNA, miR-199a-3p. This deficiency in miR-199a-3p relieves the post-transcriptional inhibition of its downstream target, Yes-associated protein 1 (YAP1). Consequently, YAP1 protein levels accumulate within the cytoplasm and nucleus. The upregulation of YAP1 serves as a critical node that activates downstream mitogenic signaling cascades. Specifically, we observed that increased YAP1 expression leads to the significant phosphorylation and activation of ERK, p38, and AKT kinases. The concerted activation of these pathways ultimately drives the pathological proliferation and migration of PASMCs, contributing to vascular remodeling. Conversely, the restoration of miR-199a-3p levels suppresses YAP1 expression, thereby inhibiting these downstream kinases and attenuating the hyper-proliferative phenotype.

## 4 Discussion

In pulmonary arterial hypertension (PAH) research, various animal and cellular models recapitulating pathological pulmonary vascular remodeling have been established. Among these, the monocrotaline (MCT)-induced and hypoxia/SU5416-induced rat models are widely recognized as classical approaches [15]. While MCT-induced PAH primarily involves acute endothelial toxicity and pulmonary inflammation, it fails to replicate the characteristic neointimal and plexiform lesions observed in human PAH [16]. In contrast, the hypoxia/SU5416 model, combining vascular endothelial growth factor receptor inhibition with chronic hypoxia, induces progressive pulmonary endothelial injury, aberrant pulmonary artery smooth muscle cell (PASMC) proliferation, and irreversible plexiform vascular lesions, thereby better mimicking human PAH pathology [17]. Consequently, the hypoxia/SU5416 model is more suitable for mechanistic studies of vascular remodeling and PAH pathogenesis. In this study, Sprague-Dawley rats subjected to three weeks of hypoxia combined with subcutaneous SU5416 administration [18] exhibited an increased right ventricular weight ratio (RV/[LV + S]) and marked pulmonary arterial wall thickening (confirmed by HE staining), indicating successful model establishment. RT-qPCR analysis of lung tissues revealed significant downregulation of miR-199a-3p in the experimental group, suggesting its potential involvement in PAH progression. PDGF-BB, a potent mitogen, drives synthetic phenotype switching in PASMCs [19], characterized by hyperproliferation, migration, apoptosis resistance, and metabolic dysregulation [20]. In this study, human PASMCs (hPASMCs) stimulated with PDGF-BB (0-40 ng/mL) exhibited maximal proliferation at 20 ng/mL. Subsequent treatment with 20 ng/mL PDGF-BB for 48 h upregulated mRNA and protein levels of synthetic markers (PCNA, CyclinD1) while downregulating contractile markers (SM22α, MYH11), confirming successful phenotypic switching. Concurrently, miR-199a-3p expression was significantly reduced in PDGF-BB-treated hPASMCs, implicating its role in this phenotypic modulation. In summary, consistent downregulation of miR-199a-3p was observed across PASMCs from PAH patients, the hypoxia/SU5416 rat model, and PDGF-BB-induced hPASMC phenotypic switching, suggesting its functional relevance in PASMC plasticity. However, the precise mechanistic contributions of miR-199a-3p require further investigation.

The miR-199a2 gene, co-embedded with miR-214 within the *DNM3os* primary transcript (NCBI Gene ID: 100,628,315), is transcribed as part of *DNM3os* and subsequently processed into mature miRNAs [21]. Consequently, transcription factors regulating *DNM3os* indirectly modulate miR-199a-3p biogenesis. Bioinformatics screening using the ENCODE database identified potential transcription factors (STAT3, RAD21, YY1, ATF2, POLR2A) binding to the *DNM3os* promoter region. Significantly, Western blot analysis revealed that PDGF-BB treatment selectively upregulated phosphorylated STAT3 (p-STAT3) levels in hPASMCs compared to other candidate factors. STAT3, a pleiotropic signaling molecule, regulates cellular proliferation, apoptosis, differentiation, and inflammation [22]. In the context of PAH, phosphorylated STAT3 (p-STAT3) drives PASMC proliferation by modulating cell cycle genes and contributes to vascular remodeling [23]. Moreover, pro-inflammatory cytokines (e.g., IL-1β) exacerbate PASMC phenotypic switching via STAT3 activation [24]. Huang et al. [25] further identified the QKI-STAT3-miR-146 b axis as a critical mediator of hypoxia-induced pulmonary vascular remodeling, wherein STAT3 enhances miR-146 b transcription to promote PASMC proliferation. However, STAT3-dependent regulation of miR-199a-3p remained unexplored prior to this study. In our experiments, adenovirus-mediated STAT3 overexpression in hPASMCs significantly reduced *DNM3os*, miR-199a2, and miR-199a-3p expression. Furthermore, dual-luciferase reporter assays demonstrated that STAT3 suppressed luciferase activity driven by the wild-type *DNM3os* promoter. Critically, this suppression was abolished by mutation of the STAT3-binding motif (TTCC to TGCG), confirming STAT3 as a transcriptional repressor of *DNM3os* and, consequently, miR-199a-3p biogenesis.

DNM3os-derived miR-199a-3p/5p has been implicated in regulating TGF-β/Smad signaling to promote pulmonary fibroblast activation and fibrosis progression [26], our study focused on its role in PASMCs. Overexpression of miR-199a-3p (via mimic transfection) in hPASMCs downregulated the protein level of the synthetic marker CyclinD1 and upregulated that of the contractile marker SM22α. Functional assays (CCK-8, scratch wound healing, and Transwell) confirmed that miR-199a-3p overexpression significantly suppressed hPASMC proliferation and migration. Conversely, knockdown of miR-199a-3p (via inhibitor transfection) enhanced CyclinD1 expression, reduced SM22α levels, and promoted proliferation and migration. Collectively, these bidirectional gain- and loss-of-function experiments demonstrate that miR-199a-3p inhibits synthetic phenotype switching in hPASMCs. In the pathway analysis section, intersection of differentially expressed genes (DEGs) in PAH-PASMCs with predicted miR-199a-3p target genes yielded 50 overlapping genes. Subsequent KEGG enrichment analysis of these 50 genes identified the MAPK and PI3K/AKT pathways as the predominant signaling cascades. To experimentally validate these KEGG predictions, we performed gain- and loss-of-function studies targeting miR-199a-3p. Western blot analysis revealed that miR-199a-3p overexpression suppressed phosphorylation of ERK and AKT, resulting in inhibition of the MAPK/ERK and PI3K/AKT pathways and thereby contributing to the maintenance of hPASMCs in a contractile phenotype. Conversely, miR-199a-3p knockdown enhanced ERK and AKT phosphorylation, activated both pathways, and induced a phenotypic transition towards the synthetic state.

## 5 Conclusions

In this study, we observed consistent downregulation of miR-199a-3p across three distinct models of pulmonary arterial hypertension (PAH): PASMCs derived from PAH patients, a hypoxia/SU5416-induced rat model, and a PDGF-BB-induced phenotypic switching model in human PASMCs (hPASMCs). Mechanistically, PDGF-BB activates STAT3, which binds specifically to the TTCCCGGAA motif within the *DNM3os* promoter, suppressing its transcription and thereby reducing miR-199a-3p expression. Furthermore, downstream pathway analysis demonstrated that miR-199a-3p inhibits abnormal hPASMC proliferation, migration, and synthetic phenotypic switching by suppressing ERK and AKT phosphorylation, consequently inhibiting activation of the MAPK/ERK and PI3K/AKT signaling pathways.

## Funding

This study was supported by the 10.13039/501100001809National Natural Science Foundation of China (No. 82070419), and Shanghai Yangpu District Medical Key Discipline Construction Fund (22YPZA08).

## CRediT authorship contribution statement

**Wen-Xia He:** Investigation, Methodology. **Yun-Jie Huang:** Investigation, Methodology. **Tian-Hong Ai:** Investigation, Methodology. **Song-Qun Huang:** Conceptualization, Formal analysis, Funding acquisition, Investigation, Investigation. **Xiao-Hua You:** Funding acquisition, Supervision. **Hong Wu:** Conceptualization, Funding acquisition, Supervision. **Xiao-Wei Song:** Conceptualization, Investigation, Supervision, Writing – original draft, Writing – review & editing.

## Declaration of competing interest

The authors declare that they have no known competing financial interests or personal relationships that could have appeared to influence the work reported in this paper.
